# Obesity-induced cofilin1 pathway dysregulation: Possible molecular links between neuroinflammation, cognitive decline, and Alzheimer's disease biomarkers

**DOI:** 10.1016/j.ibneur.2025.10.001

**Published:** 2025-10-02

**Authors:** Amsha S. Alsegiani, Bdour Alshalawi, Shaden Alzahrani, Nourah Z. Alzoman, Aliyah Almomen

**Affiliations:** Department of Pharmaceutical Chemistry, College of Pharmacy, King Saud University, PO Box 22452, Riyadh 11495, Saudi Arabia

**Keywords:** Obesity, Cofilin1, Neuroinflammation, Cognitive decline, AD biomarkers, Microbiota

## Abstract

Obesity is a growing concern globally, particularly among young populations. Cofilin1, a major actin-depolymerizing factor (ADF) isoform in the CNS, acts as a stress protein that triggers neuroinflammation and correlates with the onset of Alzheimer's disease (AD). However, its role in obesity has not yet been identified. In this study, we investigated the influence of diet-induced obesity on cofilin1 dysregulation in the brain and its correlation to neurobehavioral deficiencies, neuroinflammation, synaptic dysfunction, and AD biomarkers. Male C57BL/6 mice were fed a regular diet (control) or a high-fat diet (HFD, obese) for 7 weeks to induce obesity. Our data demonstrate that the activation of cofilin1 in the hippocampus region of HFD mice brains is mediated by Slingshot homolog-1 (SSH1) overactivation and LIM kinase-1 (LIMK1) inactivation. However, pcofilin1 is also increased, but this increase might be mediated by mechanisms other than an actin-dependent mechanism. Cofilin1 pathway dysregulation in HFD-fed mice was correlated with cognitive decline, as assessed using the Morris water maze (MWM) and Y-maze, and increased astrocytic activation protein and synaptotoxicity by downregulating pre- and post-synaptic markers. It also correlates with the significant upregulation of AD biomarkers (pTau_Ser396_) in the brain, saliva, and serum. At the systemic level, our results showed dysregulation of the gut microbiota, characterized by a ∼53 % increase in the Firmicutes: Bacteroidetes ratio in HFD-fed mice. HFD-fed mice also exhibited a significant increase in certain inflammatory and anti-inflammatory blood cytokines. Our findings suggest that active cofilin1 plays a significant role in diet-induced obesity and may become a potential therapeutic target.

## 1 Introduction

Obesity is a complex condition that has become a severe public health concern in recent years, with a high risk of morbidity and mortality (Fruh, 2017, Abdelaal et al., 2017). It is characterized by massive adipose tissue accumulation, influenced by both genetic and epigenetic factors, as well as low physical activity and long-term consumption of a high-fat diet (HFD). The prevalence of obesity has increased worldwide, especially in the younger population. According to recent studies, obesity affected almost 2.2 billion adults globally in 2022, and it is predicted that the prevalence of adult obesity will have increased from 42 % to over 54 % by 2035 (Federation, 2024). Obesity is commonly associated with sustained low-grade systemic inflammation in humans and rodents (Khanna et al., 2022). It has been suggested that the peripheral immune system produces several inflammatory markers and toxic substances related to adipose tissue, leading to chronic systemic inflammation (CSI) (Le Thuc and Garcia-Caceres, 2024). CSI is considered a silent killer that increases the risk of many other diseases (Alsegiani and Shah, 2024).

Moreover, obesity-induced CSI is a risk factor that is directly linked to the development of neuroinflammation, a hallmark of all brain diseases, and, subsequently, the development of neurodegenerative diseases (Subeta et al., 2021). Another essential component of the relationship between obesity and neuroinflammation is the gut microbiota dysbiosis through the gut-brain axis mechanism, suggesting that gut-derived metabolites might trigger stress proteins in the brain (Breton et al., 2022). In addition, many studies indicate that obesity in adults is a predisposing factor for cognitive decline and increased risk of developing Alzheimer's disease (AD) (Pedditzi et al., 2016, Dahl et al., 2013); therefore, obesity in middle age is a critical point. However, the effect of obesity on brain physiology and inflammation remains unclear, and further studies are warranted. Therefore, it is essential to understand the underlying molecular mechanism of how obesity increases the risk of dementia and AD in middle age.

Recent studies investigated an association between neuroinflammation and AD (Leng and Edison, 2021), indicating that the cofilin1 protein is one of the cellular inflammatory triggers (Bamburg and Bernstein, 2016, Rahman et al., 2014). Cofilin1 protein is an actin-associated protein that has been shown to have different roles in regulating the dynamic processes of the actin cytoskeleton through bundling, nucleating, severing, and depolymerizing filamentous actin (F-actin) (Ostrowska and Moraczewska, 2017). In addition, cofilin1 plays a vital role in mediating glial cell activation, apoptosis, synaptic impairment, and cognitive decline and is involved in the pathogenesis of AD (Bamburg and Bernstein, 2016, Alsegiani and Shah, 2020). It has been suggested that peripheral factors, such as gut microbiota dysbiosis and blood cytokines, may participate in the etiology of cofilin1 dysregulation (Alsegiani and Shah, 2024).

Several molecular mechanisms regulate cofilin1 dynamics and activity through phosphorylation and dephosphorylation processes mediated by various protein kinases and phosphatases (Van Troys et al., 2008, Mizuno, 2013). The upstream regulation exhibits the highest substrate specificity, which can affect cofilin1 activity through LIM kinase isoform 1 (LIMK1) and slingshot phosphatase isoform 1 (SSH1). LIMK1 deactivated cofilin1 through phosphorylation of the residue at Ser3 and was reactivated by dephosphorylation by SSH1. Therefore, we hypothesize that cofilin1 gradually accumulates with obesity in young adult subjects and further increases in the presence of CSI, which is induced by obesity, becoming a significant source of neuroinflammation and the development of neurodegenerative disease. Nevertheless, the cofilin1 pathway signaling in obesity has not yet been fully identified, and further research is needed to understand its dynamics.

As the life expectancy of obese people is decreasing (Abdelaal et al., 2017), it is critical to investigate the obesity-related molecular changes in the brain to develop preventive measures. Therefore, this study aims to investigate the role of cofilin1 signaling in the brains of obese mice as a neuroinflammatory mediator using the HFD-induced obesity model in young C57/B6 mice. Next, we investigate the potential correlation between dysregulation of cofilin1 signaling and neuroinflammation, cognitive decline, and the development of AD biomarkers in the brain and saliva. We also examined changes in systemic inflammatory biomarkers and alterations of gut microbiota diversity and community with obesity.

## 2 Materials and methods

### 2.1 Experimental animals

C57BL/6 male mice (4 weeks old, n = 10) were obtained from the animal house of the College of Pharmacy at King Saud University, Riyadh, Saudi Arabia. Mice were housed under standard conditions at an ambient temperature of 22°C ± 2°C and a humidity of 60 % ± 5. All animal experiments were conducted in accordance with the guidelines of the Ethical Committee for Performing Studies on Animals at King Saud University, Riyadh, Saudi Arabia, as outlined in protocol KSU-SE-19–115.

### 2.2 Experimental design

Mice were randomly divided into two groups: the healthy control and the obese group. Animals were housed in separate cages according to their assigned group to prevent cross-contamination between experimental groups. No co-housing occurred between animals from different groups. The flow chart of the experimental model is shown in Supplemental Figure 1a. The healthy control group was fed a regular diet (n = 10, 25.4 % protein, 50.3 % carbohydrate, 4.6 % fat, ∼3.5 kcal/g, Scientific Mills Co, Ltd, Riyadh, Saudi Arabia), while the obese group was fed a commercially available high-fat diet (HFD) (n = 10, 10 % protein, 20 % carbohydrates and, 60 % fat, ∼5 kcal/g, Shanghai Trens Tech Co., Ltd, Shanghai, China) for seven weeks as described in the previous studies (Yoshizaki et al., 2020, Yang et al., 2016, Xu et al., 2018). During the feeding period, the mice's body weight was tested at a fixed time once a week. Blood glucose levels were measured weekly throughout the study using tail vein blood samples, which were analyzed with an Accu-Chek glucose meter (Roche Diagnostics, Germany), to minimize potential confounding factors. At the end of the study, the mice were sacrificed, and blood and brain tissue samples were collected.

### 2.3 Neurobehavioral tests

Spatial and working memory were assessed using the Morris Water Maze (MWM) and the Y-maze tests. After week 5, mice were subjected to behavioral tests in the following order: a Y-maze test for one day and an MWM test for five successive days. All tests were tracked and analyzed using Any-Maze software [Stoelting Co, Wood Dale, IL, USA].

#### 2.3.1 Y-maze

The Y-maze test evaluated spatial working memory by measuring the spontaneous alternation rate. The mice were put in an opaque, Y-shaped maze with 40 cm of arm length, 3 cm of arm width at the bottom, 13 cm of arm width at the top, and 15 cm of wall height. The arms were symmetrically arranged at 120 angles. After being positioned in the middle of the Y-maze, the mouse had eight minutes to investigate the arena. The following formula was used to determine the rate of spontaneous alternations: [(number of alternations)/(number of total arm entries-2)]* 100.

#### 2.3.2 MWM

MWM measures spatial learning and memory. The mouse is placed in a large circular pool and is required to locate a visible or invisible platform that allows it to escape the water by using various cues. The pool is generally 150–200 cm in diameter and 45 cm deep, containing water to a depth of 26.5 cm at around 25–29 °C. The pool is fitted with a submerged platform and cues on the sides. The water will be filled and drained daily. A video camera is placed above the center of the pool to record the swimming animals and the time it takes for the animals to find the platform. The animals are continuously monitored during the time spent in the pool.

### 2.4 Saliva collection

Saliva was collected from mice as described in a previously published study (Zubeidat et al., 2022). The protein concentration of Tau proteins was measured using FastScan™ Phospho-Tau (Ser416), FastScan™ Total Tau, and PathScan® Phospho-Tau (Ser396) sandwich ELISA Kit (Cell Signaling Technology), and Aβ was measured using Aβ1–42 ELISA kit from Novus Biologicals (Centennial, CO, USA).

### 2.5 ELISA assay

Inflammatory and anti-inflammatory cytokines were measured using a Multiplex Cytokine ELISA kit protocol (MY BioSource, Multiplex Cytokine ELISA kit, San Diego, CA, USA) according to the manufacturer's instructions.

### 2.6 Western blotting (WB)

Pre-made SDS polyacrylamide gels (8–12 %) were used to analyze total protein expression levels of the hippocampus region of mouse brains using the WB technique. Proteins were transferred onto a PVDF membrane, blocked with 5 % BSA, and incubated with antibodies overnight at 4 °C. The following primary antibodies were used in the study: rabbit anti-pcofilin1, rabbit anti-cofilin1, rabbit anti-t-Tau, rabbit anti-p-Tau (ser416), rabbit anti-p-Tau (ser396), mouse anti-PSD95 and rabbit anti-SSH1 (Cell Signaling Technology, Danvers, MA, USA), rabbit anti-pSSH1(Invitrogen, Waltham, MA, USA), mouse anti-LIMK1, rabbit anti-pLIMK1, and rabbit anti-Aβ, rabbit anti-GFAP, and rabbit anti-BDNF (Abcam, Cambridge, MA, USA). Protein levels were normalized to the loading control of GAPDH. The proteins were detected using HRP-conjugated secondary antibodies: anti-rabbit or anti-mouse (Cell Signaling Technology). The images were captured using a Bio-Rad machine and analyzed using ImageJ.

### 2.7 Microbiota analysis

Fecal samples were collected freshly and directly from the mice anus and immediately stored at −80 °C until processing. Total bacterial DNA was extracted from feces using the QIAamp PowerFecal Pro DNA Kit (QIAGEN, Germantown, MD, USA) following the manufacturer's instructions. The QIIME tool was utilized to calculate diversity and analyze 16S rRNA gene sequencing, as described in a previous study (Kuczynski et al., 2011). Taxonomic classification was performed using a Naive Bayes classifier trained on the SILVA 138 database. Four α-diversity indices were used, including the ACE, Chao, number of observed OTUs, and Shannon index.

### 2.8 Statistical analysis

Statistical analysis and plotting graphs were performed using GraphPad Prism software. Results are expressed as mean ± SEM. P < 0.05 (*), P < 0.01 (**), P < 0.001 (***), and P < 0.0001 (****) were considered significant. Student unpaired t-tests and TWO-way ANOVA, followed by the Tukey test with two independent variables, were applied to compare the obese and control groups.

## 3 Results

### 3.1 Obesity and its impact on cofilin1 signaling

**Fig. 1:**
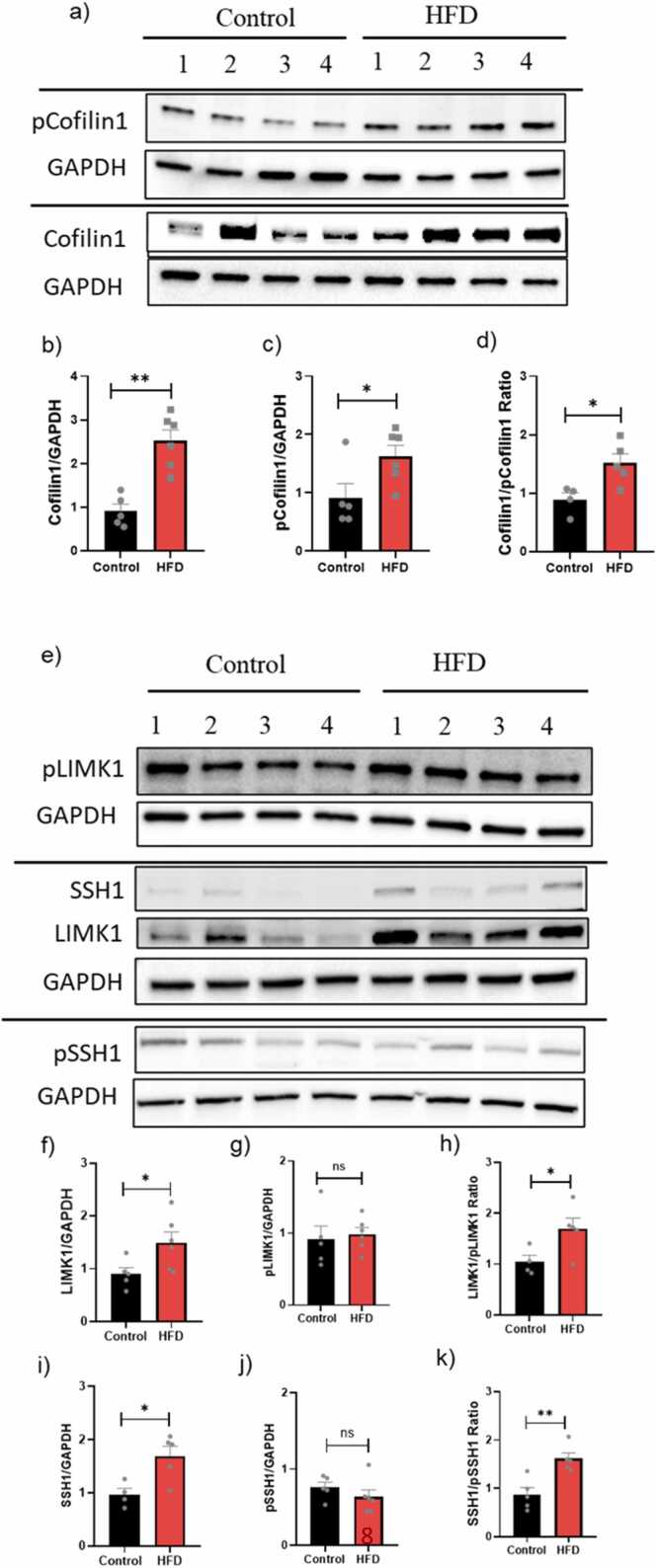
Cofilin1 pathway dysregulation in age-matched young control and HFD mice. a) Western blot analyses of total protein extracts of cofilin and pcofilin. Densitometric analyses of Western blot protein bands: b) Total Cofilin1 protein levels; c) pcofilin1 levels; d) cofilin1/pcofilin1 ratio. e) Western blot analyses of total protein extracts of LIMK1/Plimk1 and SSH1/pSSH1: f) Total LIMK1 protein levels; g) pLIMK1 levels; h) pLIMK1/LIMK1 ratio; i) Total SSH1 protein levels; j) pSSH1 levels; (k) pSSH1/SSH1 ratio. Results are expressed as mean ± SEM (n = 4–6, unpaired student's *t*-test), where P < 0.05 is considered significant. P < 0.05 (*), P < 0.01 (**), P < 0.001 (***) and P < 0.0001 (****).

To understand cofilin1-related dysregulation, we investigated upstream signaling cascades using Western blot analysis (Fig. 1a,e). The expression of total cofilin1 and pcofilin1 signaling in HFD mice was significantly increased by 150 % (*P < 0.05) and 125 % (**P < 0.01), respectively (Fig. 1b,c). The ratio of cofilin1 to p-cofilin1 significantly showed a ∼50 % increase in the cofilin1 dephosphorylation expression, suggesting cofilin1 activation (Fig. 1d). The upstream pathway also showed an increase in SSH1 and LIMK1 in HFD mice (*P < 0.05) (Fig. 1f, i), with no significant changes observed in pLIMK1 and pSSH1(Fig. 1g,j). In addition, LIMK1/pLIMK1 (*P < 0.05) and SSH1/pSSH1 ratios (**P < 0.01) were significantly increased compared to the control mice (Fig. 1h,k).

### 3.2 Obesity and its impact on cognitive functions

**Fig. 2:**
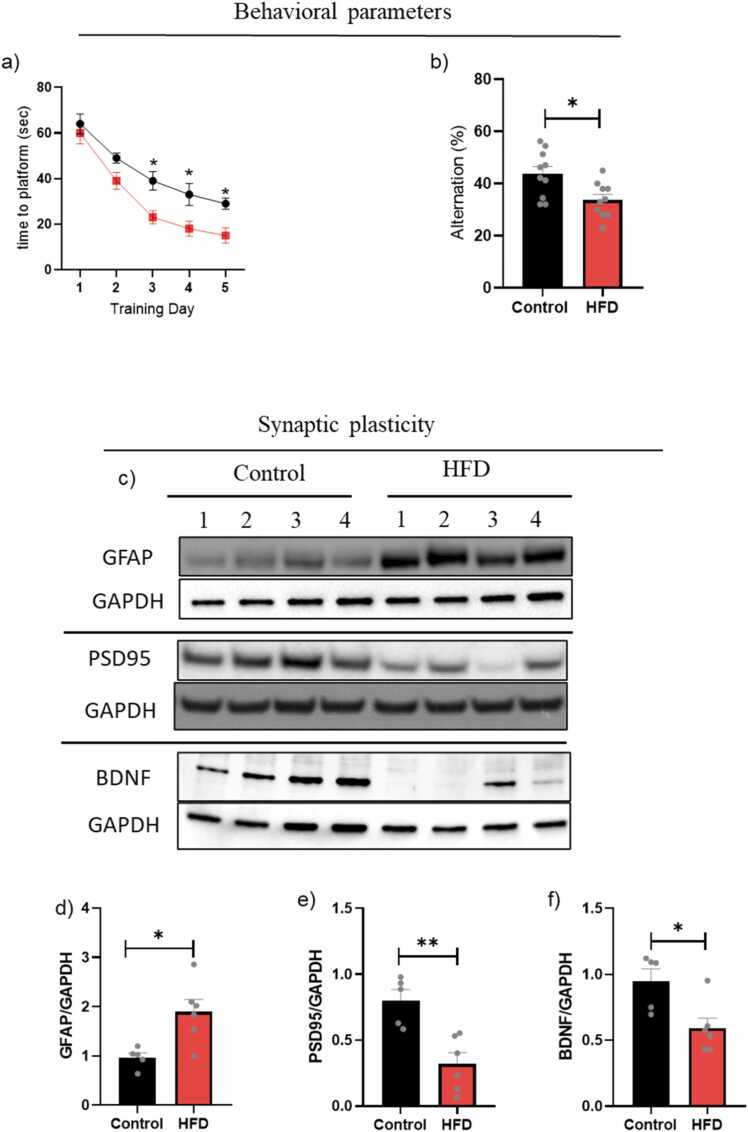
Neuroinflammation, synaptic plasticity, and neurobehavioral outcomes in age-matched young control and HFD mice. Neurobehavioral outcomes: a) MWM test, b) Y-maze test, data are expressed (n = 10). c) Densitometric analyses of Western blot protein bands (n = 4–6): d) GFAP protein levels; e) PSD95 protein levels; f) BDNF protein levels. Results are expressed as mean ± SEM (n = 8–10, unpaired student's *t*-test), where P < 0.05 is considered significant. P < 0.05 (*), P < 0.01 (**), P < 0.001 (***) and P < 0.0001 (****).

A schematic of the study design is presented in the Supplemental Figure 1a. The mice exhibited a 20–40 % increase in body weight after HFD in week four and continuously increased until the end of the experiments without an increase in blood sugar (Supplemental Figure 1 b,c). Neurobehavioral parameters (Morris water (MWM) and Y-maze) and body weight measurements were assessed before (baseline) and after HFD, compared to the control group. Our results showed that obese mice performed significantly worse in the cognition tests. Obese mice exhibited substantially longer times to explore the platform in the MWM on days 3, 4, and 5 (*P < 0.05) (Fig. 2a and Supplemental Figure 5). Furthermore, obese mice exhibited a decreased spontaneous alternation rate in the Y-maze compared to the control group (*P < 0.05) (Fig. 2b).

### 3.3 Obesity and its impact on neuroinflammation and synaptic dysfunction

Glial cells, especially microglia and astrocytes, play an essential role in controlling inflammation in the CNS. Glial fibrillary acidic protein (GFAP) expression levels were evaluated using WB to explore astrocytic activation (Fig. 2c). Our results showed a significant increase in GFAP (*p < 0.05) in the brains of HFD mice (Fig. 2d), indicating astrogliosis and neuroinflammation.

Based on results showing cognitive dysfunction in HFD mice in MWM and Y-maze tests, we assessed the effect of obesity on synapse density by evaluating the levels of two synaptic markers, post-synaptic density protein-95 (PSD-95) and Brain-derived neurotrophic factor (BDNF) (Fig. 2c). Our results showed that obese mice lose synaptic function significantly by ∼60 % and ∼40 %, decreasing the expression of PSD95 (**p < 0.01) and BDNF (*p < 0.05) proteins, respectively (Fig. 2e,f).

### 3.4 Obesity and its impact on AD biomarkers

To examine the correlation between cofilin1 dysregulation and AD biomarkers, WB analysis of brain tissues and a non-invasive saliva detection test were performed.

#### 3.4.1 AD biomarkers in the brain

**Fig. 3:**
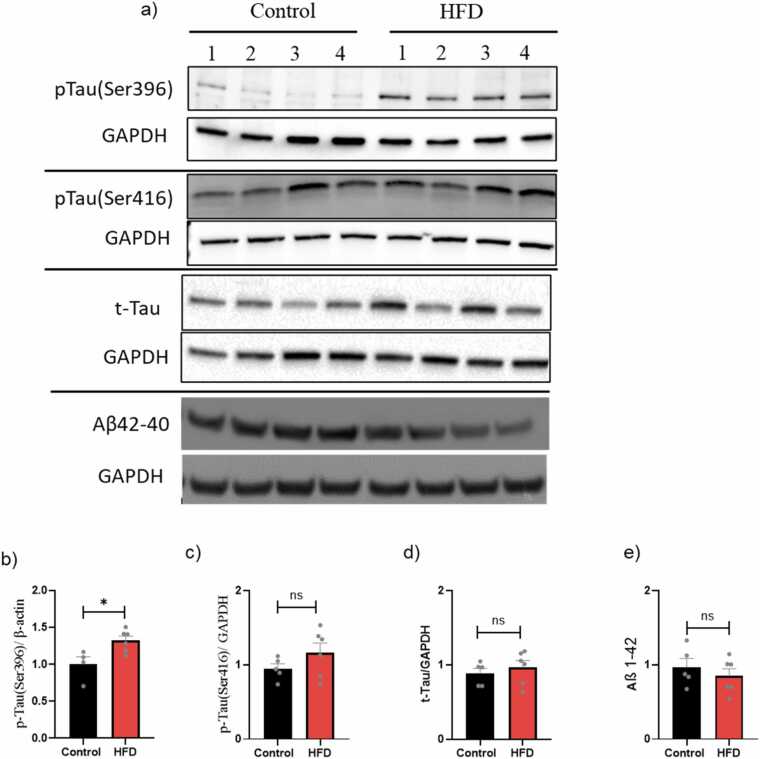
Brain AD protein biomarkers in age-matched young control and HFD mice. a) Densitometric analyses of Western blot protein bands: b) p-tau(396) protein levels; c) p-tau(396) protein levels; d) p-tau(396) protein levels; e) t-Tau protein levels. Results are expressed as mean ± SEM (n = 4–6, unpaired student's *t*-test), where P < 0.05 is considered significant. P < 0.05 (*), P < 0.01 (**), P < 0.001 (***) and P < 0.0001 (****).

Based on a previous study, two p-tau forms at sites Ser416 and Ser396 are used to detect phosphorylated tau in mouse brains (Fig. 3a) (Leng and Edison, 2021). The HFD mice group showed significant upregulation of pTau(Ser396) (*P < 0.05) compared with non-obese mice (Fig. 3b). Furthermore, all the tested groups showed non-substantial changes in the pTau(Ser416), t-tau expression, and Aβ1–42 (Fig. 3c,d,e).

#### 3.4.2 AD biomarkers in serum and saliva

**Fig. 4:**
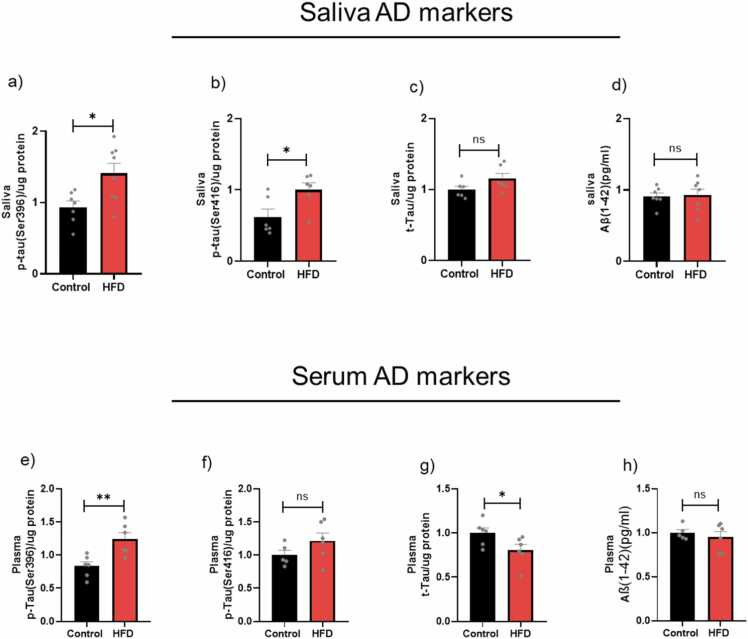
Salivary and plasma AD protein biomarkers in age-matched young control and HFD mice. Salivary ELISA protein expression: a) p-tau(396) protein levels; b) p-tau(396) protein levels; c) p-tau(396) protein levels; d) t-Tau protein levels. Plasma ELISA protein expression: e) p-tau(396) protein levels; f) p-tau(396) protein levels; g) p-tau(396) protein levels; h) t-Tau protein levels. Results are expressed as mean ± SEM (n = 4–6, unpaired student's *t*-test), where P < 0.05 is considered significant. P < 0.05 (*), P < 0.01 (**), P < 0.001 (***) and P < 0.0001 (****).

A non-invasive diagnostic test using saliva and serum was performed to investigate the pre-symptomatic phase of AD biomarker levels as peripheral biomarkers and to determine whether they reflect brain biomarkers. There was an apparent increase of p-tau (ser396) levels in both saliva and serum by ∼41 % (* P < 0.05) and ∼50 % (** P < 0.01), respectively (Fig. 4a,e). In addition, there was a significant increase in p-tau (ser396) levels in saliva with no difference in serum (Fig. 4d,f). No significant differences were observed in Aβ levels of salivary and serum (Fig. 4d,h). Interestingly, the serum t-tau levels were significantly decreased in the HFD group compared to control mice (Fig. 4g).

### 3.5 Obesity and its impact on gut microbiota alteration

**Fig. 5:**
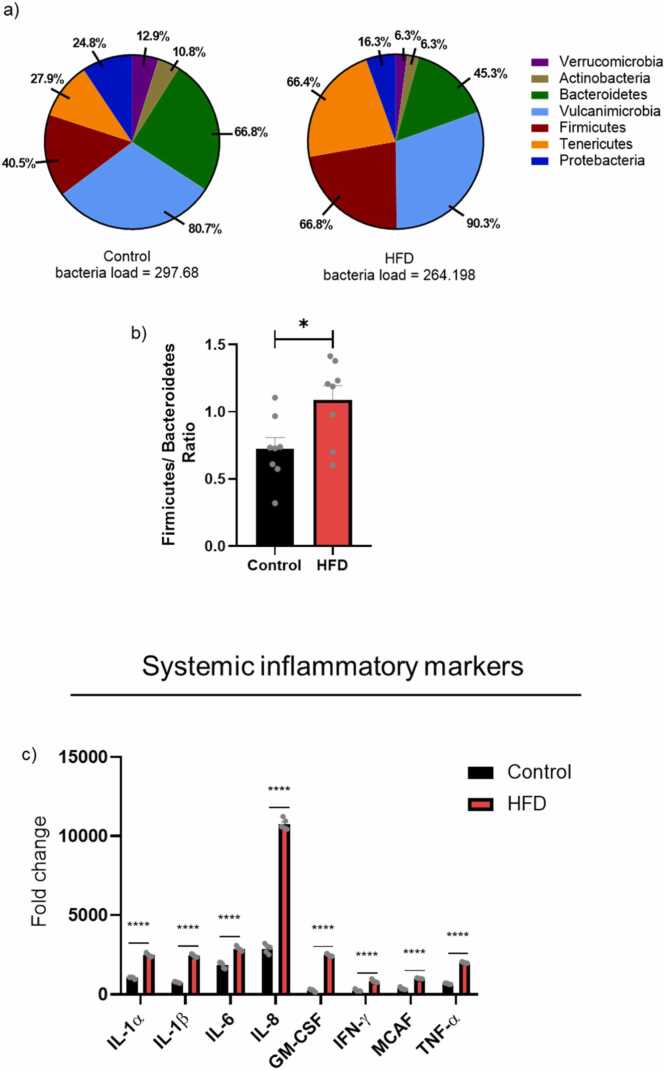
Relative abundance of microbial community and systemic inflammation in age-matched young control and HFD mice. Gut microbiota composition was analyzed with 16S rRNA sequencing(n = 8): a) the relative abundance of the different bacterial groups, the most highly abundant bacterial groups are indicated; b) Firmicutes/ Bacteroidetes ratio (n = 8). C) fold change in plasma ELISA protein expression of different pro- and anti-inflammatory biomarkers (n = 8). Results are expressed as mean ± SEM (n = 8, unpaired student's *t*-test), where P < 0.05 is considered significant. P < 0.05 (*), P < 0.01 (**), P < 0.001 (***), and P < 0.0001 (****).

To illustrate the differences in the gut microbiota in obese mice, 16S rRNA gene sequencing analysis was conducted for gut bacterial composition and diversity. The most highly abundant bacterial groups are indicated. As shown in Fig. 5a, five genera were predominant in fecal samples from obese and control mice, including Bacteroidetes, Vulcanimicrobia, Firmicutes, Tenericutes, and Proteobacteria. Our results showed that the relative abundance of Vulcanimicrobia, Firmicutes, and Tenericutes was increased in obese mice compared with the control group (10 %, 38 %, and 58 %, respectively). In contrast, the Proteobacteria and Bacteroidetes relative abundance was decreased by ∼50 %. Furthermore, our results showed dysregulation of the gut microbiota by increasing the Firmicutes to Bacteroidetes ratio in HFD mice by ∼53 % compared with the control group (Fig. 5b).

### 3.6 Obesity and its impact on systemic inflammatory biomarkers

To investigate whether altars in the gut microbiota community correlate with the circulation system, inflammatory and anti-inflammatory cytokine markers in plasma were measured using ELISA (Fig. 5c). Our results showed that both inflammatory and anti-inflammatory cytokines were significantly elevated in obese mice compared to non-obese, including IL-6, IL-8, GM-CSF, TNF-α, IL-1β, IFN-γ, and MCAF. Interestingly, IL-8 increased 10,000-fold in obese mice***.***

## 4 Discussion

In the present study, we evaluated the cofilin1 expression in young-adult obese mice for the first time using an HFD-induced obesity model. We also investigated the role of cofilin1 in mediating neuroinflammation and neurotoxicity, as well as its correlation with the upregulation of AD biomarkers and systemic inflammation, by examining gut microbiota dysbiosis and blood cytokines. It was found that the cofilin1 pathway in the brain was dysregulated by activating the cofilin1 protein through the overactivation of SSH1 and the inactivation of LIMK1. The study outcomes suggest that cofilin1 activation might play a role in neuroinflammation, cognitive decline, and increased risk of developing AD.

Our results are in agreement with those previously published on feeding HFD in C75/B6 mice (Yoshizaki et al., 2020, Li et al., 2020), which significantly affects body weight gain and becomes evident after four weeks (Supplemental Figure 1b). Since obesity is characterized by various metabolic abnormalities, such as elevated blood glucose levels, we observed a non-significant increase in blood glucose levels in obese mice (Supplemental Figure 1c), which means our obese mouse model did not develop diabetic disease. Therefore, we performed our study without confounding factors related to diabetes in cofilin1 dysregulation. However, the potential for additional metabolic disorders to contribute to neuroinflammation and cofilin1 dysregulation is not excluded, even in the absence of elevated glucose levels, such as insulin resistance and altered lipid profiles.

Disruption of cofilin1 dynamics in the brain has been linked to the pathophysiology of many neurodegenerative diseases and aging (Alsegiani and Shah, 2024, Barone et al., 2014). Cofilin1-induced neuroinflammation is further supported by upstream modulators through LIMK1 and SSH1 proteins. Noticeably, LIMK1 is inactivated by dephosphorylation of the LIMK1 residue at Thr508, whereas total SSH1 activation is regulated by dephosphorylation at residue Ser978 (Bravo-Cordero et al., 2013). To our knowledge, no study has examined the effects of HFD-induced obesity on the dysregulation of the cofilin1 pathway. Our results showed that the dysregulation of the cofilin1 pathway occurred in the brains of HFD mice, resulting from the activation of cofilin1 due to the overactivation of SSH1 and the inactivation of LIMK1. This finding was confirmed by increased SSH1/pSSH1 and LIMK1/pLIMK1 ratio. We also found an increase in inactive cofilin1 expression; however, the active cofilin1 protein level was significantly higher than that of inactive cofilin1. This suggests that the upregulation of inactive cofilin1 may play a partial role in obesity by blocking actin remodeling and could be involved in molecular mechanisms beyond actin-dependent pathways. This could include the phosphatidylinositol 4,5-bisphosphate (PIP2) signaling pathway, which inhibits cofilin1 activity by competing with actin at its binding site, and the ubiquitin-proteasome pathway, which is regulated by cofilin1 phosphorylation at the Y68 residue (Zhao et al., 2010, Yoo et al., 2010). An increase in both active and inactive cofilin1 suggests that upstream signaling pathways controlling cofilin1 activity are imbalanced. This imbalance affects actin remodeling, leading to cytoskeletal disruption, synaptic dysfunction, and neurodegeneration. This dysregulation also contributes to neuroinflammation, as abnormal actin remodeling influences microglial activation and inflammatory responses.

Cofilin1 is regulated by an upstream phosphatase mediator, SSH1, that has a high affinity for dephosphorylation and activates cofilin1 in response to stress signals, such as reactive oxygen species (ROS) accumulation and ATP depletion (Mizuno, 2013, Kim et al., 2009, Huang et al., 2008). In accordance with this, we observed cofilin1 dephosphorylation in HFD mice brains through SSH1 overexpression. It was recognized that the SSH1 protein regulates cofilin1 activity by dephosphorylation of the cofilin1 residue at Ser3 (Kurita et al., 2008). Additionally, SSH1 can dephosphorylate the LIMK1 protein and reduce its enzymatic activity in response to cofilin1 (Soosairajah et al., 2005). Consequently, our results also showed a concurrent increase in LIMK1 expression, concomitant with increased SSH1 expression and cofilin1 dephosphorylation, suggesting SSH1 inhibits LIMK1 activation. These findings suggest that SSH1 overexpression, rather than LIMK1 inactivation, is the predominant mechanism underlying cofilin dysregulation in our model. Taken together, an increase in SSH1 and LIMK1 contributes to cofilin1 activation, which could result from neuroinflammation.

Previous preclinical evidence has investigated the mechanisms by which HFD-induced neuroinflammation, impairment in synaptic plasticity, and decline in cognitive performance occur (Valladolid-Acebes, 2024, Sobesky et al., 2014). For example, hippocampal leptin resistance, hyperinsulinemia, and elevated glucocorticoid levels are associated with cognitive decline and synaptic decline in adult HFD rodent models. In addition, cofilin1 activity is involved in various signaling mechanisms that contribute to synaptic dysfunction and are related to impairments in neurobehavioral parameters (Alsegiani and Shah, 2024, Ben Zablah et al., 2020, Alhadidi et al., 2018). Similarly, our results showed neuroinflammation, synaptic decline, and impairment in spatial learning and short-term memory in HFD mice compared with the healthy control group. Our results also consistently indicate that cognitive decline and synaptic dysfunction in the HFD model were correlated with dysregulation of the cofilin1 pathway, suggesting that long-term CSI in obesity affects hippocampal function. However, other potential pathways, including cytokine signaling, glial activation, and the gut-brain/gut-liver-brain axis, may also be involved in the dysregulation of cofilin1 and its role in the pathogenesis of obesity-induced neuroinflammation and cognitive decline (Zeng et al., 2024, Yan et al., 2023).

The secondary aim of the present study was to examine whether cofilin1 dysregulation in HFD mice is correlated with the abnormality of AD biomarkers in the brain, saliva, and serum. Previous clinical studies have confirmed that obesity-related brain changes and cognitive decline resemble those of AD patients (Morys et al., 2023, Deckers et al., 2017). Furthermore, it has been found that the overexpression of active cofilin1 increases p-tau expression by inhibiting tau-induced microtubule assembly, forming rod-shaped aggregation as an early stage of AD development (Rahman et al., 2014, Woo et al., 2019). Based on our data, the significant increase in p-tau (Ser396) is concurrent with a substantial increase in active cofilin1, suggesting that it is regulated by mechanisms that extend to active cofilin1 upregulation. In addition, CSI, which is linked to obesity, can cause Aβ deposition and tau hyperphosphorylation in rodents (Wang et al., 2018, Thygesen et al., 2018). Another clinical study reported that obesity in adults is associated with the accumulation of Aβ as an early marker of AD (Dolatshahi et al., 2024). Conversely, we found no changes in Aβ levels in our mice. Based on these findings, obesity in young adults are at a higher risk of developing AD, which could start early in the accumulation of p-tau through mechanisms that might be extended to active cofilin1 upregulation. However, our results indicate cofilin1 dysregulation in the HFD mice model, consistent with alterations in AD biomarkers. While this suggests a possible connection, our study does not directly demonstrate a role for cofilin1 in AD progression. We therefore propose this as a potential mechanism to be tested in future work.

Recently, peripheral biomarkers have been used to identify AD, including salivary glands and blood. Saliva, a non-invasive fluid, contains Tau and Aβ proteins secreted by acinar epithelial cells in the salivary glands. AD biomarkers have been reported to increase in AD patients, attracting attention for the detection of diagnostic biomarkers (Bermejo-Pareja et al., 2010, Shi et al., 2011). We observed a simultaneous increase in both salivary and serum p-tau (Ser396) levels, consistent with their presence in the brain, but no changes were observed in p-tau (Ser416) and Aβ proteins. Additionally, the t-Tau level decreased in serum. These results are consistent with previous studies that reported increased p-tau (Ser396) expression levels in saliva and CSF samples from aging-related CSI and AD patients (Alsegiani and Shah, 2024, Leslie et al., 2021). Obesity in adults is considered a high-risk factor for developing AD. Additionally, AD biomarkers can be detected peripherally in both saliva and serum. Overall, p-tau (ser396) could be a non-invasive biomarker for the early detection of AD in obese subjects.

Obese mice exhibited general metabolic and gut inflammation, correlated with high levels of inflammatory markers in the circulatory system, as reported in various studies (Shemtov et al., 2023). In many studies, obese subjects have been shown to exhibit differences in fecal microbiota diversity and composition compared to non-obese individuals (Alili et al., 2021, Shahinozzaman et al., 2021). In parallel, we observed that HFD mice had lowered fecal bacterial diversity and community (Barengolts, 2016). In addition, at the phylum level, our results are similar to those of studies that have shown genetically obese and HFD mice to be enriched in Firmicutes and depleted in Actinobacteria and Bacteroidetes (Shahinozzaman et al., 2021, Davis, 2016). The alteration of gut microbiota increases the risk of CSI by triggering innate immunity and elevating blood inflammatory cytokines (Khanna et al., 2022, Le Thuc and Garcia-Caceres, 2024). Obesity-related gut microbiota dysfunction can negatively impact cognition by interfering with the gut–brain axis (Agusti et al., 2018, Olsthoorn et al., 2021, Yarahmadi et al., 2024). Alterations in microbial composition can trigger systemic inflammation, impair nutrient metabolism, and influence neurotransmitter production, all of which can affect brain function. These changes are associated with cognitive deficits, such as memory impairment and diminished executive function, highlighting a connection between obesity-related microbiota imbalances and reduced mental performance. While our findings suggest a potential link between gut microbiota alterations and changes in cofilin1 signaling, we acknowledge that this relationship remains correlative in nature. The observed associations between microbial shifts and cofilin1 dysregulation support the hypothesis that microbiota-derived signals may influence cofilin1 dynamics; however, direct mechanistic evidence is currently lacking. Therefore, we propose this as a possible pathway that warrants further investigation. Future studies will be necessary to determine whether targeting the gut microbiota can directly modulate cofilin1 activity and mitigate neurodegenerative processes.

During gut dysbiosis, bacteria can secrete large amounts of byproducts, such as lipopolysaccharides (LPS), which contribute to the excessive production of pro-inflammatory cytokines, including interleukin-6 (IL-6) (Biagi et al., 2010). This bacterial secretion can induce neuroinflammation by exacerbating BBB permeability and dopaminergic loss (Zeng et al., 2024, Wu et al., 2021). Similarly, our results showed that the surge in systemic inflammatory biomarkers in obese mice was represented by an increase in M1-proinflammatory cytokines, a decrease in M2-anti-inflammatory cytokines, and an elevation in myeloid-derived suppressor cells (MDSCs). These critical findings were observed through an increase in plasma levels of IL-6, IL-8, IL-1α, IL-1β, TNF-α, GM-CSF, MCAF, and IFN-γ. These results are consistent with the idea that cofilin1 dysregulation is correlated with gut microbiota alteration and systemic inflammation in obese mice.

## 5 Conclusions and study limitations

**Fig. 6:**
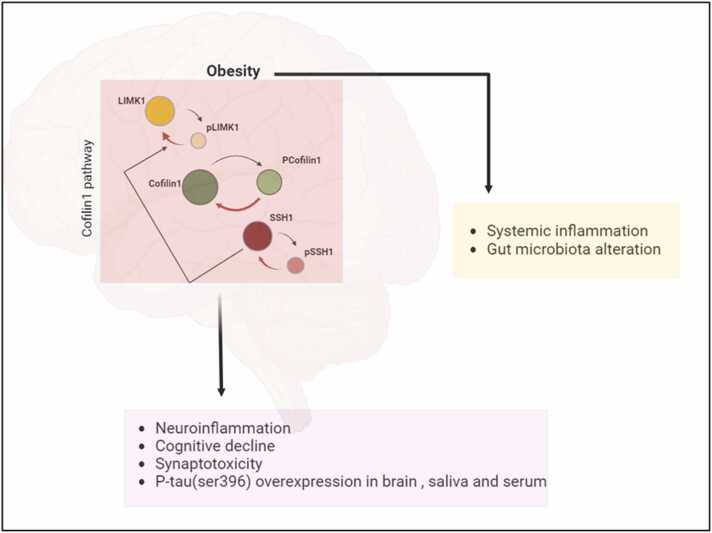
Cofilin1 pathway in obesity. The cofilin1 pathway plays an essential role in glial activation and is correlated with reduced levels of PSD95 and BDNF, leading to cognitive impairments and increased AD markers. In addition, obesity is associated with systemic inflammation and gut microbiota alteration.

In this study, we investigated the effects of HFD on cofilin1 pathway dysregulation in the brain as inflammatory markers in relation to a healthy diet. Cofilin1 was overexpressed due to an increase in SSH1 and LMK1, which may be a consequence of neuroinflammation. Moreover, the neuroinflammatory response to cofilin1 activation in adult mice with obesity correlates with impaired neurobehavioral and synaptic function, elevated AD protein markers, activation of astrocyte cells, alterations in gut microbiota, and heightened inflammatory cytokine levels in the circulation (Fig. 6). These results provide new insights into cofilin1 as a target in obesity that might help modulate obesity complications in the CNS. While our findings are suggestive, several limitations must be acknowledged. These include the need for larger sample sizes to strengthen statistical power, more detailed analyses of cofilin1 activation states, and the exploration of additional molecular pathways that may mediate the observed effects. Furthermore, we focused exclusively on middle-aged male animals, which limits the generalizability of our results. Future research should examine potential sex- and age-related differences in the relationship between obesity and cofilin1 signaling. Additionally, confounding factors such as insulin resistance and dysregulated lipid metabolism should be systematically assessed for their contribution to neuroinflammation and cofilin1-related mechanisms. Ultimately, identifying the specific, obesity-driven inflammatory pathways that directly regulate cofilin1 activity will be crucial for understanding the causal links and translating these findings into therapeutic strategies for both animal models and humans.

## Author contributions

**AA, AAA**, and **NA** participated in the research design; **BA** and **SA** conducted experiments. **AA, AAA, BA**, and **SA** contributed to new reagents or analytic tools; **AA** and **AAA** performed data analysis, and **AA** and **AAA** wrote and contributed to the manuscript.

## CRediT authorship contribution statement

**Bdour Alshalawi:** Methodology. **Nourah Z. Alzoman:** Writing – original draft, Visualization, Supervision. **Shaden Alzahrani:** Methodology. **Aliyah Almomen:** Writing – review & editing, Writing – original draft, Visualization, Validation, Supervision, Resources, Project administration, Methodology, Investigation, Funding acquisition, Formal analysis, Data curation, Conceptualization. **Alsegiani Amsha:** Writing – review & editing, Writing – original draft, Validation, Supervision, Project administration, Methodology, Investigation, Data curation, Conceptualization.

## Institutional review board statement

All animal experiments strictly followed the guidelines of the Ethical Committee for Performing Studies on Animals at King Saud University, Riyadh, Saudi Arabia, as outlined in protocol number KSU-SE-19–13.

## Financial support

This research was supported by funding from the Ongoing Researcher Funding Program (ORF-2025-1125), 10.13039/501100002383King Saud University, Riyadh, Saudi Arabia.

## Conflicts of interest

The authors declare that they have no conflicts of interest.

## Data Availability

The authors declare that all data supporting the findings of this study are available within the paper and its Supplemental Material.
