# Supplementary material for: Obesity-induced cofilin1 pathway dysregulation: Possible molecular links between neuroinflammation, cognitive decline, and Alzheimer's disease biomarkers
Source: IBRO Neurosci Rep. 2025 Oct 2;19:699–708. doi: 10.1016/j.ibneur.2025.10.001 (PMC12538084; doi:10.1016/j.ibneur.2025.10.001)
Supplement: Supplementary file 1 — Supplementary material [file mmc1.docx]

**Obesity-Induced Cofilin1 Dysregulation: Molecular Link Between Neuroinflammation, Cognitive Decline, and Alzheimer's Pathology**

**Amsha S. Alsegiani ^1,†^, Bdour Alshalawi^1^, Shaden Alzahrani^1^,** [**Nourah Z Al-Zoman**](https://pubmed.ncbi.nlm.nih.gov/?term=Al-Zoman+NZ&cauthor_id=39049887)**^1^, and Aliyah Almomen ^1,†,^***

^1^ Department of Pharmaceutical Chemistry, College of Pharmacy, King Saud University, PO Box 22452, Riyadh 11495, Saudi Arabia; [Aalsegiani@ksu.edu.sa](mailto:Aalsegiani@ksu.edu.sa); [nalzoman@ksu.edu.sa](mailto:nalzoman@ksu.edu.sa); [alalmomen@ksu.edu.sa](mailto:alalmomen@ksu.edu.sa)

*****Correspondence: [alalmomen@ksu.edu.sa](mailto:alalmomen@ksu.edu.sa)

† These authors contributed equally to this work


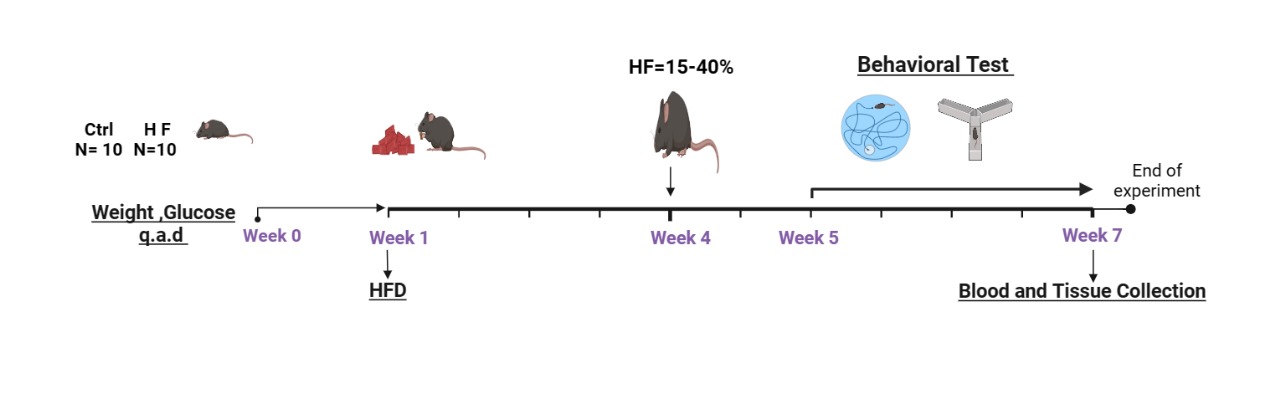

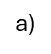


**Supplemental Figure 1:** Experimental design. a) The flow chart of the experimental model, b) body weight, c) blood glucose level. Results are expressed as mean ± SEM, and *P<0.05 was considered significant.


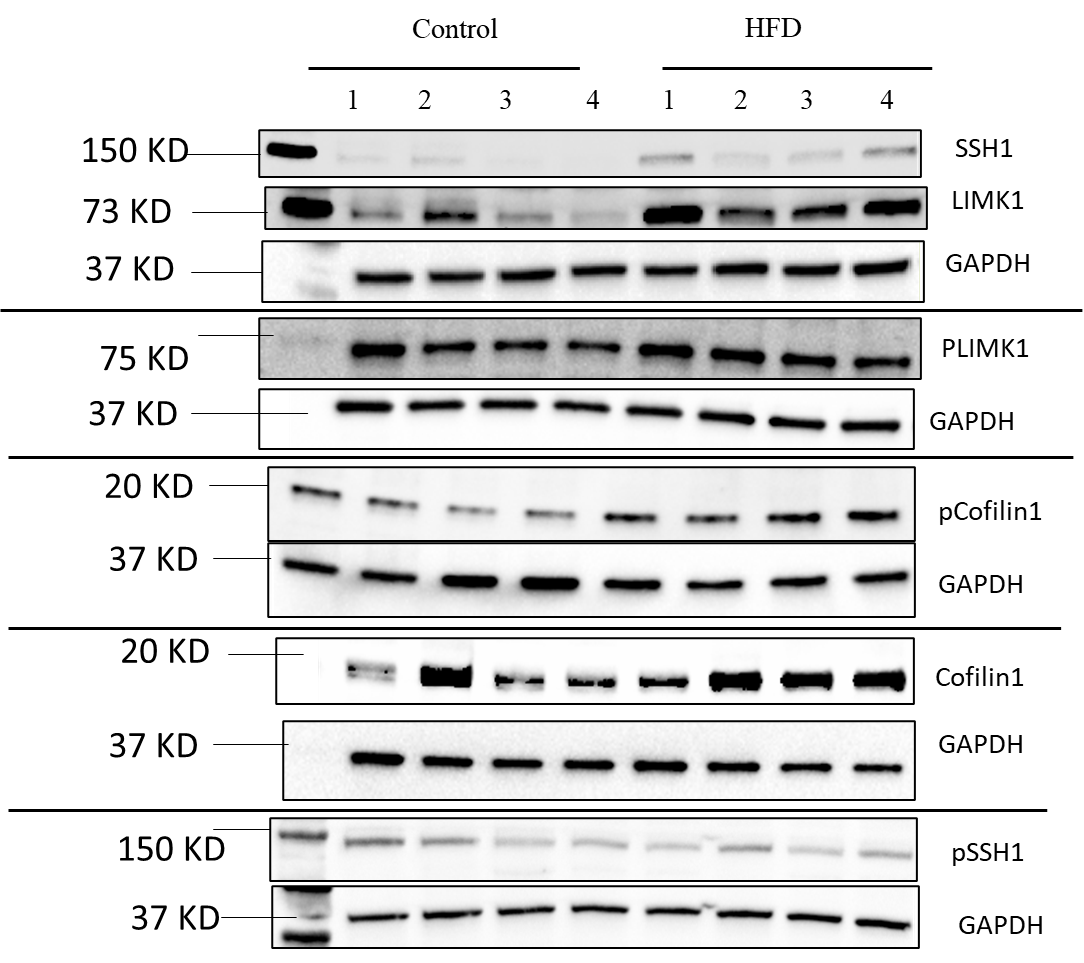


**Supplemental Figure 2:** Original western blot for Figure 1 showing the bands with molecular weight markers.


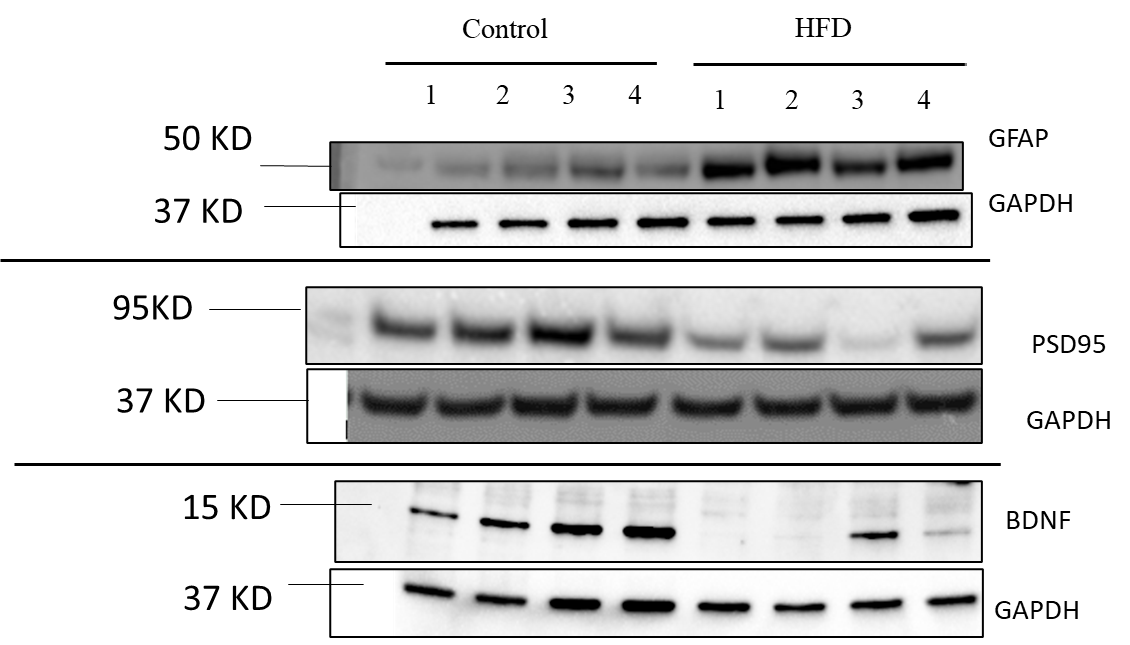


**Supplemental Figure 3:** Original western blot for Figure 2 showing the bands with molecular weight markers.


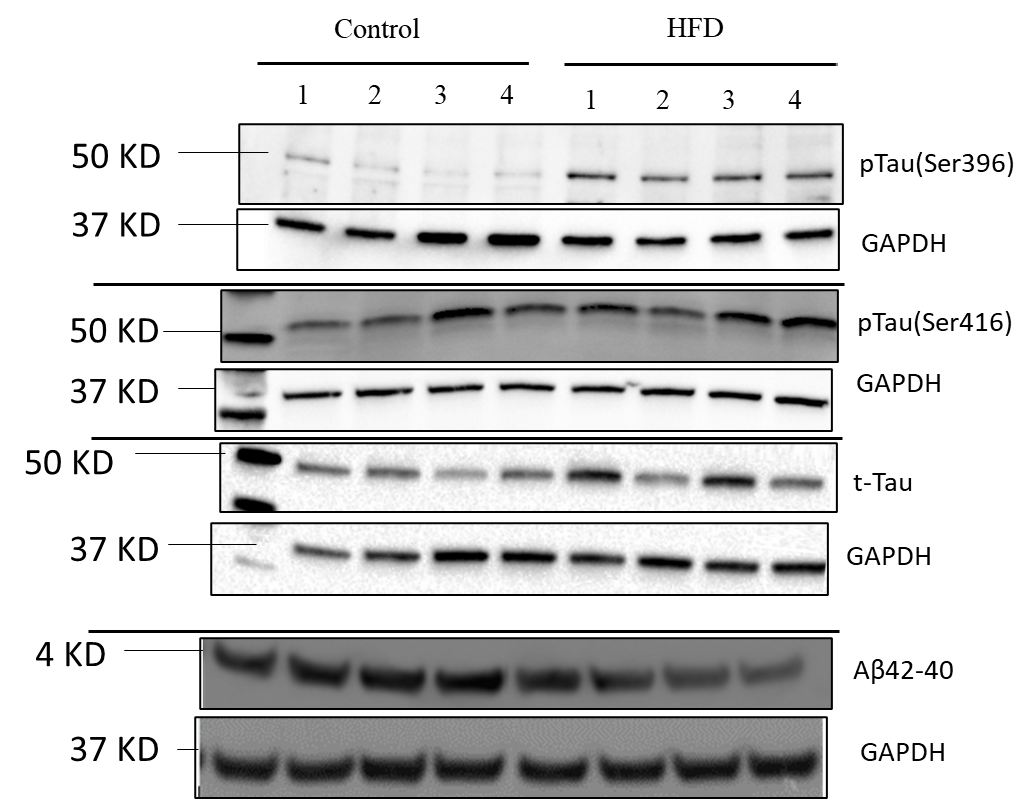


**Supplemental Figure 4:** Original western blot for Figure 3 showing the bands with molecular weight markers.


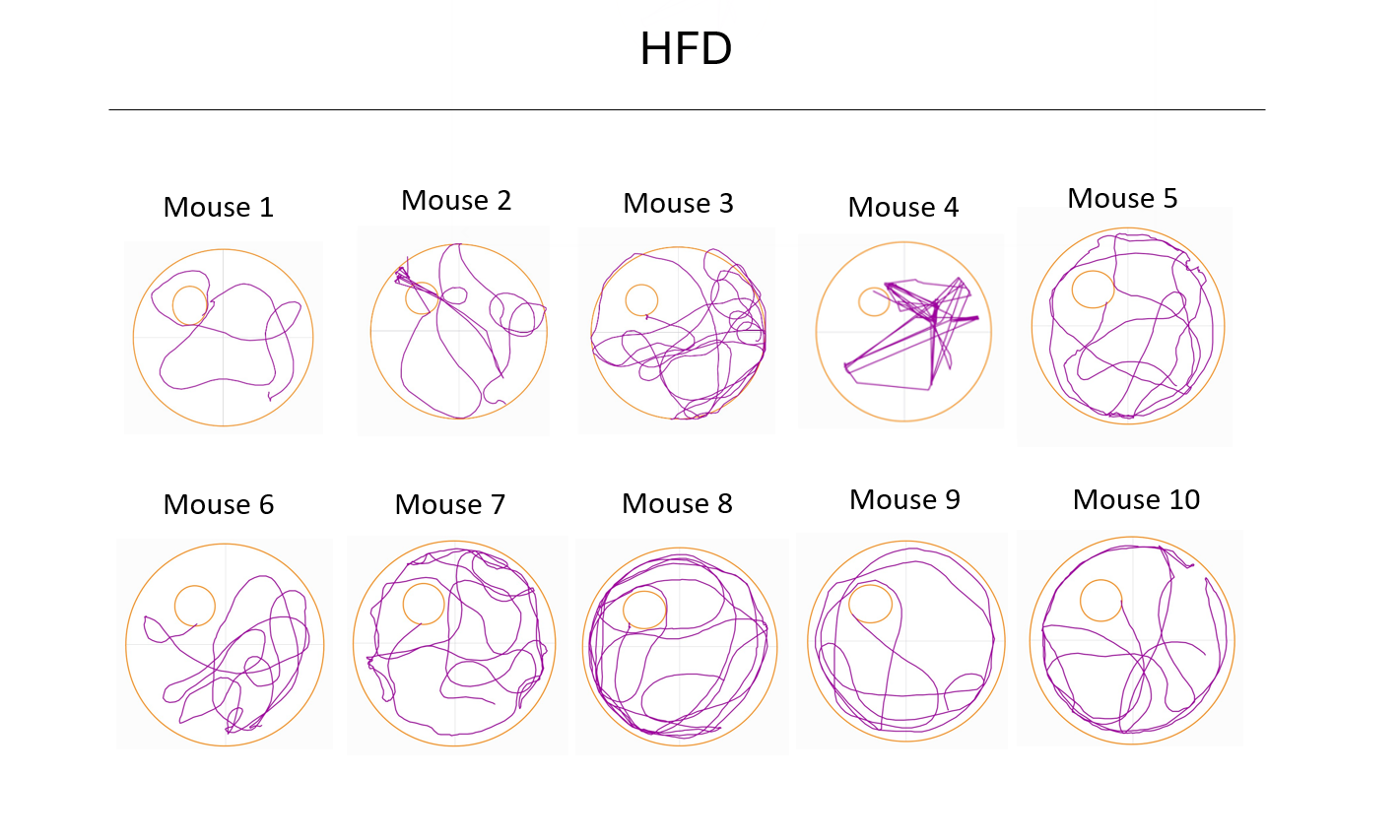

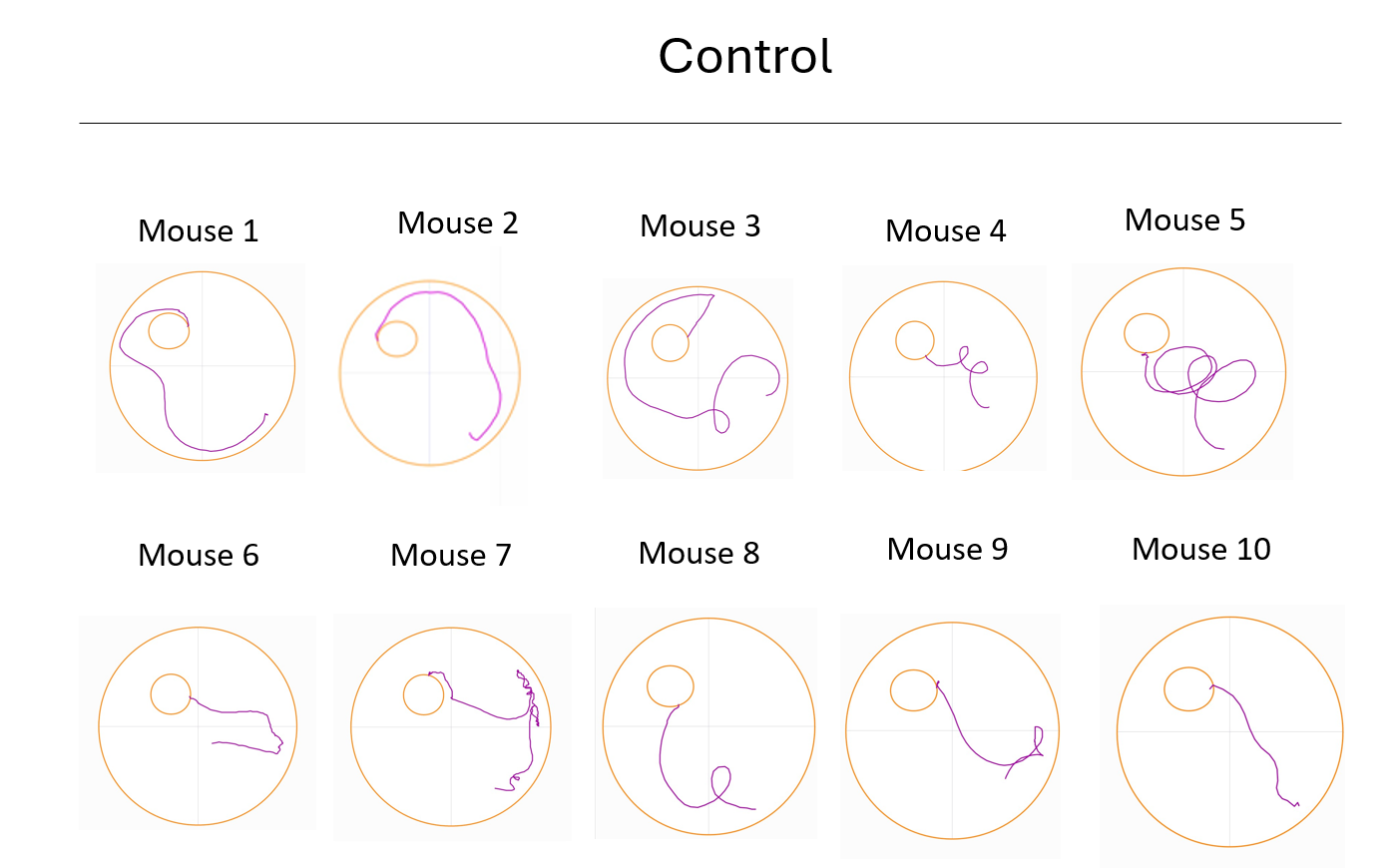


**Supplemental Figure 5:** Representative swim path trajectories of control and HFD-induced obese mice during the Morris water maze (MWM) trials.
